# Metavac-RSV mucosal bivalent vaccine candidate protects cotton rats against pneumoviruses and is produced using serum-free cell culture in bioreactor

**DOI:** 10.1038/s41541-025-01231-9

**Published:** 2025-08-22

**Authors:** Caroline Chupin, Pauline Brun, Marjorie Ray, Chloé Mialon, Maëlle Reitano, Aurélien Traversier, Emilie Laurent, Abdelghafar Goumaidi, Julien Fouret, Stéphane Paul, Marina Boukhvalova, Kevin Yim, Jorge Blanco, Marie-Eve Hamelin, Guy Boivin, Manuel Rosa-Calatrava, Julia Dubois

**Affiliations:** 1 International Research Laboratory RESPIVIR France - Canada, Centre Hospitalier Universitaire de Québec-Université Laval, Québec, Canada ; Centre International de Recherche en Infectiologie, Faculté de Médecine RTH Laennec, Université Claude Bernard Lyon 1, Université de Lyon, INSERM, CNRS, ENS de Lyon, 69008 Lyon, France; 2 CIRI, Centre International de Recherche en Infectiologie, Team VirPath, Univ Lyon, Inserm, U1111, Université Claude Bernard Lyon 1, CNRS, UMR5308, ENS de Lyon, F-15 69007 Lyon, France; 3 Vaxxel, 43 Boulevard du onze novembre 1918, 69100 Villeurbanne, France; 4 Virnext, Faculté de Médecine RTH Laennec, Université Claude Bernard Lyon 1, Université de Lyon, 69008 Lyon, France; 5 Nexomis, Faculté de Médecine RTH Laennec, Université Claude Bernard Lyon 1, Université de Lyon, 69008 Lyon, France; 6 CIRI, Centre International de Recherche en Infectiologie, Team GIMAP, Université Claude Bernard Lyon 1, INSERM U1111, CNRS UMR5308, ENS Lyon, Université Jean Monnet Saint-Etienne, Saint-Etienne, France; 7 Sigmovir Biosystems, Inc., 9610 Medical Center Drive, Suite 100, Rockville, MD 20850 USA; 8 Centre de Recherche en Infectiologie of the Centre Hospitalier Universitaire de Québec and Université Laval, Laval, QC G1V 4G2 Canada

**Keywords:** Vaccines, Live attenuated vaccines, Infectious diseases

## Abstract

Respiratory syncytial virus (RSV) and human metapneumovirus (HMPV) are the main etiologic agents of viral bronchiolitis and pneumonia in children and the elderly. As live-attenuated vaccines (LAV) can stimulate robust mucosal and cellular responses, we previously engineered an HMPV-based bivalent LAV Metavac®-RSV candidate and reported its capacity to protect mice against HMPV and RSV challenges after intranasal delivery. To progress towards clinical development, we identified a GMP-grade Vero cell platform as permissive and efficient to produce high yields of functional Metavac®-RSV, expressing both RSV and HMPV F antigen after several passages. Metavac®-RSV protected cotton rats against both HMPV and RSV challenges, significantly reducing viral replication in the respiratory airways and inducing high titers of neutralizing antibodies. Finally, we identified process parameters to scale-up the production process of Metavac®-RSV using Vero cells cultivated on microcarriers in a 2 L single-use stirred-tank bioreactor, with a scalable upstream production process amenable to industrial manufacturing.

## Introduction

Among acute respiratory tract infections (ARI), human pneumoviruses, which include human respiratory syncytial virus (RSV) and human metapneumovirus (HMPV), represent a major cause of diseases, especially for children, older adults and immunocompromised individuals^1–4^. Before the COVID-19 pandemic, RSV was responsible for 33 million acute respiratory infections among children under 5 years old, with 11% of these children being admitted to the hospital^5^. For older adults, RSV was responsible for 1.5 million of ARI leading to 15% of hospitalization^6^. Notably, HMPV also affects children between 1 and 3 years of age^7,8^ and more than 90% of children are infected during their first 5 years of age^9^. The COVID-19 pandemic had a major impact on seasonality and prevalence of pneumoviruses in children and adult populations, with 2.4-fold more cases during the 2022–2023 season than before the pandemic^10–15^.

Over the past few decades, several immunization strategies were assessed to prevent pneumovirus diseases, such as monoclonal antibodies, recombinant protein vaccines^16,17^, live-attenuated vaccines (LAVs)^18,19^, and more recently, mRNA candidates^20–22^. Based on knowledge about the immunogenicity of the RSV-F protein, most of the development of protein and mRNA vaccine candidates focused on this antigen in pre-fusion conformation (pre-F)^23,24^. Hence, since 2023, two recombinant RSV pre-F proteins vaccines (GSK’s Arexvy® and Pfizer’s Abrysvo®) and one mRNA vaccine encoding RSV pre-F protein (Moderna’s mRESVIA®) have been approved by the FDA and the EMA for the prevention of RSV infection in the elderly and for the passive immunization of newborns (only for Abrysvo®) *via* vaccination of pregnant women^12,25–27^. Awaiting an approved pediatric vaccine, two prophylactic monoclonal antibodies, targeting the RSV-F protein, are also used for passive protection in the first year of child exposure: palivizumab (Synagis®, AstraZeneca) and nirsevimab (Beyfortus®, Sanofi Pasteur)^28,29^. LAV strategies are particularly suitable for pediatric immunization, as they elicit both humoral and mucosal immunity by mimicking natural viral replication routes and do not require adjuvants^30^. Thus, some pneumovirus-based LAV candidates, such as M2-2, NS2, and G/SH gene-deleted RSVs, as well as G/SH-deleted HMPVs, have shown promising results in preclinical models and some of them are currently in the clinical development phase^19,31–33^. This approach differs from formalin-inactivated pneumovirus-based vaccines, which have been associated with vaccine-enhanced respiratory disease (ERD) in the past^34^.

In this context, we have previously described that the bivalent vaccine candidate Metavac®-RSV, derived from the C-85473 HMPV strain with cleavage of the SH gene, expresses both HMPV and RSV-F proteins and exhibits genetic stability after several passages in LLC-MK2 cells^32,33,35,36^. When administered to BALB/c mice by intranasal route, Metavac®-RSV replicates in the lungs of mice with reduced lung inflammatory score and protects against subsequent RSV and HMPV challenges while inducing strong IgG and broad RSV and HMPV neutralizing antibody responses^36^.

Since its discovery in 2001, the HMPV virus has been commonly cultivated on LLC-MK2 cells to obtain a high yield of infectious particles but other adherent cell lines have also shown to be permissive to infection, such as Vero or HEp-2^1,37,38^. In the perspective of bioproduction process development, we previously validated the DuckCelt®-T17 cells, an avian cell line cultivated in suspension without serum, for the production of functional monovalent Metavac® candidate^35^. However, Metavac®-RSV did not propagate sufficiently in DuckCelt®-T17 cells to meet production yield objectives for industrial development (data not shown). On the other hand, Vero cells are a well-established cell line in viral vaccine manufacturing as they are used for the production of several FDA-approved vaccines such as polio, rabies, rotavirus, and smallpox^39,40^. Among its advantages, these cells have a wide susceptibility to many viruses, and manufacturers have a long-term experience with the Vero cell culture. Indeed, since Vero cells isolation in 1962^41^, many derivations from the original cell line exist, among which are serum-free adapted cells, in agreement with regulatory requirements^39^.

In this study, we evaluated culture parameters for Metavac®-RSV LAV production in a GMP-grade Vero cell line, and we characterized the resulting product in terms of efficacy of antigen expression and in vivo protection. Firstly, we validated the permissiveness and productivity of Vero cells for Metavac®-RSV cultivated in static culture, and we assessed after several cell passages its genetic stability and the efficient expression of antigenic RSV and HMPV-F proteins. Furthermore, Metavac®-RSV produced in Vero cells was evaluated in vivo in the cotton rat model and demonstrated its ability to induce neutralizing antibody responses and protect animals against HMPV and RSV challenges. This preclinical in vivo model has been widely used to evaluate the protective efficacy of multiple vaccine candidates and has been described as the most permissive small animal model to study RSV infections and to evaluate the risk of ERD^42–44^. Afterward, we identified infection parameters of Vero cells cultivated on microcarriers in agitated culture at a small-scale working volume and optimized process parameters to produce high titers of infectious Metavac®-RSV in a 2 L stirred-tank bioreactor. Altogether, these studies underscore the potential of Metavac®-RSV as a promising bivalent LAV candidate to prevent pneumovirus disease and the use of the Vero cell line as an appropriate and scalable production platform amenable to industrial manufacturing for clinical development.

## Results

### Vero cells are permissive to Metavac®-RSV and appropriate for viral production

**Fig. 1:**
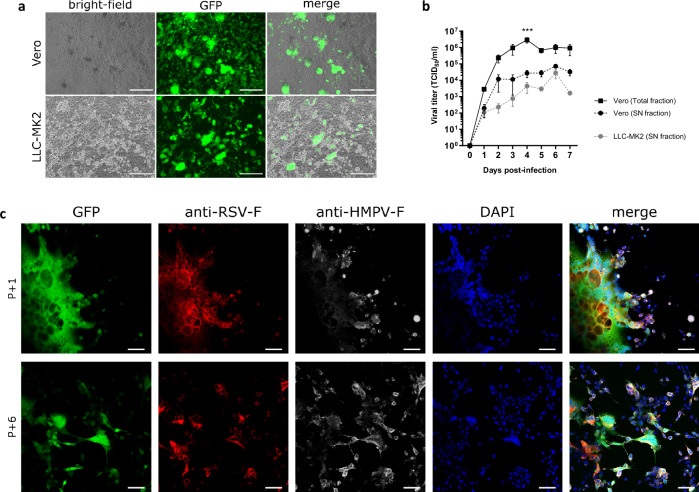
Vero cells production capacity of Metavac®-RSV. **a**, **b** Confluent Vero or LLC-MK2 cells monolayers were infected with Metavac®-RSV at an MOI of 0.01. **a** Pictures of representative cytopathic effects (CPEs) of Metavac®-RSV on these cells were taken at 4 days post-infection (dpi) in bright-field and fluorescent microscopy. Scale bar = 250 μm. **b** Viral titers were measured in samples of supernatants (SN fraction) or Total fraction (cells and supernatants) as TCID_50_/mL. Results represent the mean of three experimental replicates (mean ± SEM). ***p < 0.001 when compared to Vero SN fraction using two-way ANOVA and Dunnett post test. **c** LLC-MK2 **c**ells were infected with Metavac®-RSV produced by 1 or 6 successive cell passages on Vero cells. Infected cells were stained at 3 dpi with Palivizumab (anti-RSV-F, red), HMPV24 mAb (anti-HMPV-F, white), and DAPI (nucleus, blue). Merged fluorescent signals are represented in yellow. Images of representative CPEs were taken (×40 magnification). Scale bar = 50 μm.

We evaluated the permissiveness of a GMP-compliant Vero cell line, purchased from Nuvonis Technologies GmbH, to Metavac®-RSV virus by infecting static cell monolayers in comparison with LLC-MK2 cells. At 4 days post-infection (dpi), expression of GFP reporter protein showed that Metavac®-RSV propagated in Vero cells monolayer with well-developed cytopathogenic effects, and harbored expected hyperfusogenic phenotype, to a similar extent as that in LLC-MK2 cells (Fig. 1a). In order to compare virus growth between these two cell lines, we then conducted replication kinetics assays over a 7-day period. At 6 dpi, we obtained a maximum virus titer for Metavac®-RSV of 7.1 × 10^4^ TCID_50_/mL in the supernatant (SN) of Vero cells, compared to 2.8 × 10^4^ TCID_50_/mL for LLC-MK2 cells (Fig. 1b). With a bioprocess development perspective, we then asked whether higher amounts of infectious viral particles could be harvested from the cell fraction. Hence, viral titration was also performed from Vero cells scratched into culture medium (cells and supernatant, i.e., total fraction) to compare production yields to the supernatant (SN) fraction. Interestingly, it appears that the viral titer was significantly higher in the total fraction of Vero cells than in the SN fraction at 4 dpi with titers of 2.9 × 10^6^ TCID_50_/mL and 2.7 × 10^4^ TCID_50_/mL, respectively (Fig. 1b).

To evaluate the stability of Metavac®-RSV candidate over multiple replication cycles, Metavac®-RSV was then serially-passaged on Vero cells six times, and the complete viral genome was sequenced (Table 1). After six passages (P + 6), we identified four non-synonymous mutations (E294G, Q434H, S466R, and K518E) in the HMPV-F gene and three others (K65R, K551T, and *575Y) in the RSV-F gene (Table 1). Interestingly, these mutations had a maximal frequency of 0.25, and none were previously described as major modifications in antigenic sites^45–47^. The two more frequent mutations, E294G and Q434H, in the HMPV-F gene could be associated with the pH-dependent phenotype induced by in vitro culture adaptation^48–50^. We also described one mutation resulting in the loss of a stop codon in the RSV-F gene that extends the intracellular domain by nine amino acids (aa), as already described in our previous study using LLC-MK2 cells^36^. Besides these seven genomic changes, the 17 other mutations in Metavac®-RSV (Supplementary Table S1) were found with a frequency of less than 10%, except for two in the HMPV-M-protein gene (D31G and Y66C) that are not positioned in a critical area^51,52^. To confirm that these mutations have no impact on the expression of F proteins or on infectivity, we performed immunostaining on LLC-MK2 cells infected with Metavac®-RSV produced from one and six passages on Vero cells. Together with the GFP expression pattern showing the conservation of the hyperfusogenic phenotype, immunostaining with anti-RSV-F (Palivizumab) and anti-HMPV-F (HMPV24) monoclonal antibodies (mAbs) highlighted that both F proteins were well recognized by the corresponding mAbs and that the proteins were still expressed in cells infected by Metavac®-RSV (Fig. 1c).

**Table 1:** Genome stability of Metavac®-RSV produced in Vero cells

CDS	Nucleic			Codon		Amino acid			Alternative frequency		
Name	Position^(a)^	Ref	Alt	Ref	Alt	Position^(b)^	Ref	Alt	P + 1	P + 4 ^(c)^	P + 6 ^(c)^
HMPV-F	4694	A	G	GAA	GGA	294	E	G	-	0.12	0.25
	5115	G	T	CAG	CAT	434	Q	H	-	0.09	0.19
	5211	C	A	AGC	AGA	466	S	R	-	-	0.07
	5365	A	G	AAG	GAG	518	K	E	-	-	0.04
RSV-F	5668	A	G	AAG	AGG	65	K	R	-	-	0.07
	7126	A	C	AAG	ACG	551	K	T	-	-	0.04
	7199	A	T	TAA	TAT	575	*	YLIKNKVN*	0.09	0.19	0.24

List of single non-synonymous mutations identified in genes encoding sequence (CDS) for F proteins of Metavac®-RSV. Table details the reference base (“Ref”) and the variant base (“Alt”) at that specific nucleotide or amino acid position that represent mutations over a minimum frequency of 0.03 and short indels with a minimum frequency of 0.1.

^a^Starting from the beginning of Metavac®-RSV genome.

^b^First position is the start codon of each protein sequence.

^c^Mean frequency of two independent lineages.

Altogether, these results show that a GMP-compliant Vero cell line is appropriate to efficiently produce Metavac®-RSV, while underscoring stable expression of both RSV and HMPV antigenic F proteins with regard to minor genomic substitutions that are common for RNA viruses.

### Metavac®-RSV produced in Vero cells protects against HMPV challenge in cotton rats

In order to ensure that the vaccine candidate Metavac®-RSV produced in Vero cells preserved its vaccine properties, similarly to those previously reported in a mouse model^36^, we evaluated its performance in a cotton rat model.

First, Metavac®-RSV was administered by intranasal route (IN) to 5 cotton rats at a dose of 3.7 × 10^5^ PFU per animal to measure replication and virus-induced inflammation. Metavac®-RSV replicated efficiently in the respiratory tract of cotton rats and showed attenuation, with infectious virus titers at 5 dpi being significantly lower in the lungs and noses of Metavac®-RSV-inoculated animals compared to animals infected with HMPV (10^5^ PFU per animal) (Supplementary Fig. S1a). Both infections induced pulmonary inflammation, with Metavac®-RSV causing a slightly higher and more uniform increase in pulmonary histopathology scores compared to primary HMPV infection (Supplementary Fig. S1b). Elevated expression of the Th1-type cytokine IFNγ and the chemokine MIP-1α was detected in Metavac®-RSV-inoculated cotton rats, while no significant change in the expression of Th2-type cytokines IL-5 and IL-13 was noted in Metavac®-RSV-inoculated cotton rats compared to animals infected with HMPV. Moreover, expression of another Th2-type cytokine, IL-4, was lower in Metavac®-RSV-inoculated animals (Supplementary Fig. S1c).

In the main efficacy study, young female and male cotton rats were immunized with IN Metavac®-RSV or Metavac® dose of 3.7 × 10^5^ PFU per animal, IN boosted with the same vaccine dose 4 weeks later, and challenged after 3 more weeks with HMPV at 10^5^ PFU per animal. As an alternative vaccination, five animals were immunized twice with formalin-inactivated (FI)-HMPV via IM route and infected with HMPV, or infected with HMPV and challenged with HMPV again 7 weeks later.

**Fig. 2:**
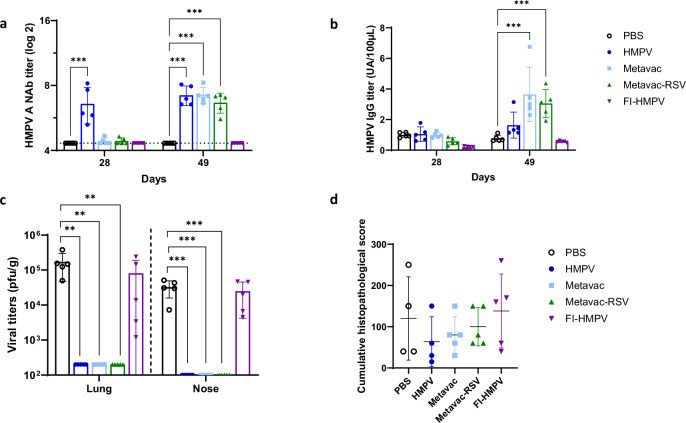
Efficacy and immunogenicity of Metavac®-RSV candidate produced in Vero cells against HMPV in cotton rats. Cotton rats were inoculated with PBS, HMPV, Metavac® or Metavac®-RSV by IN route, or IM immunized with FI-HMPV. **a** Circulating HMPV A neutralizing antibodies (NAbs) titers were measured from serum harvested at 28 and 49 days. The dotted lines represent the detection threshold. **b** Titers of circulating HMPV-IgGs were measured by ELISA assay. **c**, **d** Animals were challenged with HMPV on day 49 and, on day 54, lungs and nasal tissue were collected. **c** Pulmonary and nasal viral titers were measured in tissue homogenates. **d** Histopathology scores were evaluated in fixed lung tissue, and the mean cumulative scores are represented. Results are shown as mean ± SD (*n* = 4–5). **p < 0.01, ***p < 0.001, when compared to PBS-vaccinated animals (ANOVA and Dunnett post test).

Infection with HMPV and two-times immunization with Metavac® and Metavac®-RSV led to a similarly increased levels of neutralizing antibody (NAbs) titers against HMPV A by day 49, with titers of 7.2 log_2_, 7.2 log_2_, and 6.6 log_2_, respectively (Fig. 2a). Similarly, Metavac® vaccinated groups showed a significant induction of HMPV-specific IgG at day 49 with IgG titers increased by 4.7- or 3.9-fold compared to PBS-immunized animals, for Metavac® or Metavac®-RSV vaccination respectively (Fig. 2b). In contrast, groups immunized with PBS or FI-HMPV showed no detectable NAb or HMPV IgG (Fig. 2a, b).

We then evaluated the ability of Metavac® vaccinations to reduce viral replication and infection-induced inflammation. Five days post-challenge, no infectious HMPV virus was detected in the lungs of animals IN-immunized with HMPV, Metavac® or Metavac®-RSV whereas PBS- and FI-HMPV-immunized animals showed HMPV titers of 1.7 × 10^5^ or 8.0 × 10^4^ PFU/g of lungs, respectively (Fig. 2c). Moreover, HMPV viral loads in the nose were under the limit of detection for HMPV, Metavac® and Metavac®-RSV vaccinated groups, whereas PBS- and FI-HMPV-immunized groups showed mean viral titers of 3.2 × 10^4^ PFU/g and 2.5 × 10^4^ PFU/g, respectively (Fig. 2c). Concerning inflammatory response, no significant differences in cumulative histopathology score were measured in lungs 5 days after viral challenge between each group (Fig. 2d). Animals IN-immunized with HMPV once or twice with Metavac® or Metavac®-RSV had a tendency to show a lower level peribronchiolitis compared to PBS- or FI-HMPV-immunized, HMPV-infected animals (data not shown). Pulmonary cytokine response after HMPV challenge of Metavac®-RSV vaccinated animals was generally comparable to or lower than that seen in other vaccinated groups or animals infected twice with HMPV (Supplementary Fig. S2a).

In summary, we have shown that vaccination of cotton rats with Metavac®-RSV induced the production of high levels of neutralizing antibodies against HMPV after two immunizations, resulting in no infectious virus detectable in the lungs or noses of animals after HMPV challenge and a controlled inflammatory response.

### Metavac®-RSV produced in Vero confers protection against RSV challenge in cotton rats

We then sought to evaluate Metavac®-RSV protection against RSV challenge. Similar to the previous in vivo protocol, young female and male cotton rats were immunized with an IN Metavac®-RSV dose of 3.7 × 10^5^ PFU per animal or with FI-RSV injection via IM route, boosted with the same vaccine dose 4 weeks later, and challenged after 3 more weeks with RSV (10^5^ PFU/animal). Control animals were infected with RSV once, and challenged with RSV again 7 weeks later. All animals were sacrificed 5 days after the challenge.

**Fig. 3:**
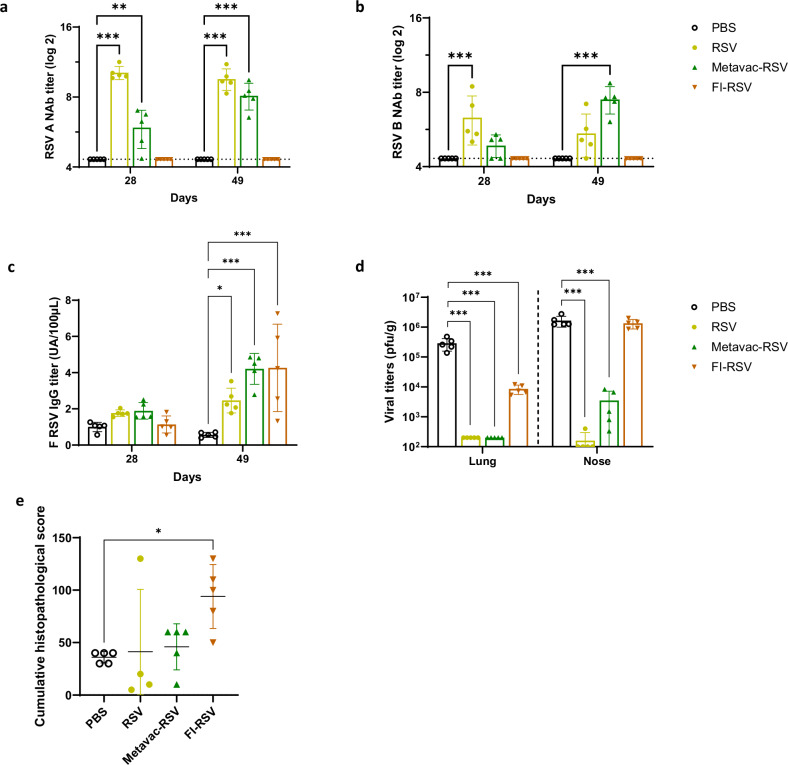
Efficacy and immunogenicity of Metavac®-RSV candidate produced in Vero cells against RSV in cotton rats. Cotton rats were inoculated with PBS, RSV or Metavac®-RSV by IN administration, or immunized IM with FI-RSV. **a**, **b** Circulating NAbs titers against RSV A (**a**) or RSV B (**b**) were measured in serum harvested at 28 and 49 days. The dotted lines represent the detection threshold. **c** Titers of circulating IgGs specific to RSV A2 F protein was measured by ELISA assay. **d**, **e** Animals were challenged with RSV at day 49 and, at day 54, lungs and nasal tissue were collected. **d** Pulmonary and nasal viral titers were measured from tissue homogenates. **e** Histopathology scores were evaluated in fixed lung tissue, and the mean cumulative scores are represented. Results are shown as mean ± SD (*n* = 4–5). *p < 0.05, **p < 0.01, ***p < 0.001 compared to PBS-inoculated animals (ANOVA and Dunnett post test).

We first measured anti-RSV NAbs response after two vaccinations and compared it to the NAbs response in animals immunized by infection with RSV, which had the highest level of RSV A NAbs on day 28 (10.2 log_2_) and on day 49 (9.5 log_2_) (Fig. 3a). Immunization with Metavac®-RSV induced a seroconversion of vaccinated cotton rats with mild production of RSV A NAb on day 28 with a titer of 5.9 log_2_ (Fig. 3a). Three weeks after the boost, RSV A NAb titer of Metavac®-RSV-vaccinated animals increased to 8.1 log_2_ by day 49, with comparable level to the RSV-immunized group (Fig. 3a). The induction of RSV B NAbs followed a similar pattern and resulted on day 49 with the highest RSV B NAbs titers of 7.5 log_2_ for Metavac®-RSV immunized animals (Fig. 3b). RSV- and Metavac®-RSV immunized-animals also showed a significant increase in binding IgG levels by day 49, compared to PBS-inoculated animals (Fig. 3c). In contrast, no RSV A or B NAbs were detectable in FI-RSV-immunized animals (Fig. 3a, b), whereas similar levels of IgG antibodies were detected compared to other vaccinated groups (Fig. 3c).

We then investigated whether Metavac®-RSV vaccination was efficacious against RSV replication in vivo. In contrast to the PBS-immunized group, which showed RSV titer of 2.85 × 10^5^ PFU/g in lungs 5 days post-challenge, there was no detectable infectious virus in the lungs of animals IN-immunized with RSV or Metavac®-RSV (Fig. 3d). RSV viral loads in the nose were under the limit of detection for the RSV-vaccinated group, and significantly reduced in Metavac®-RSV vaccinated animals, with a titer of 3.5 × 10^3^ PFU/g compared to 1.6 × 10^6^ PFU/g in the PBS group (Fig. 3d). Conversely, compared to the PBS-vaccinated group, FI-RSV-immunized animals showed a limited reduction of RSV titers in the lungs, with 8.5 × 10^3^ PFU/g detected, and no reduction in nasal viral load, with a titer of 1.3 × 10^6^ PFU/g (Fig. 3d).

Concerning the inflammatory response, the highest level of cumulative histopathology was detected in FI-RSV-immunized animals (Fig. 3e), whereas animals immunized with RSV or with Metavac®-RSV showed a similar level of cumulative histopathology compared to PBS-immunized animals. Interestingly, the Th2 inflammatory response measured by IL-4 and IL-13 levels was similar for PBS, RSV and Metavac®-RSV vaccinated groups, whereas FI-RSV vaccination tended to induce increased levels of these cytokines (Supplementary Fig. S2b). Expression of Th1-type cytokine IFNγ or chemokines MCP-1 and MIP-1α in Metavac®-RSV-immunized animals was similar to, or slightly elevated compared to the other groups of animals in the study (Supplementary Fig. S2b).

To conclude, we have demonstrated that cotton rats immunized with Metavac®-RSV produced a broad antibody response against RSV A and B after two immunizations, with a significant reduction of RSV replication in the upper and lower airways after RSV challenge, contrasting with FI-RSV vaccination, which showed limited protection against viral infection and an increased pulmonary histopathology and Th2-type cytokine expression.

### Identification of infection parameters for Metavac®-RSV production in Vero cells on microcarriers

Since Metavac®-RSV vaccine candidate produced in Vero cells has been shown to protect cotton rats against both HMPV and RSV, we then sought to develop a scalable production process of the LAV candidate in industrial manufacturing perspective. As a first step, we adapted our cell culture parameters to cultivate Vero cells on Cytodex1 microcarriers in a small agitated volume in TubeSpin® (data not shown). We then evaluated infection parameters such as frequency and concentration of trypsin, multiplicity of infection (MOI), and volume at the time of inoculation to identify the best conditions for Metavac®-RSV production at the small scale.

**Fig. 4:**
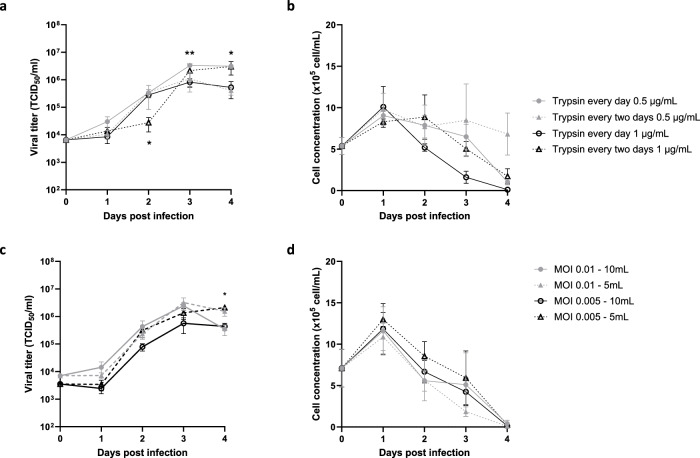
Identification of infection parameters for the production of Metavac®-RSV in TubeSpin®. Vero cells were seeded at 1 × 10^5^ cells/mL with Cytodex1 in TubeSpin® and grown for 4 days before virus inoculation. **a**, **b** After cells infection with Metavac®-RSV at an MOI of 0.01, a regimen of trypsin supplementation was evaluated comparing the effect of trypsin addition every day or every 2 days at a final concentration of 0.5 or 1 µg/mL on viral titration (**a**) and cell growth (**b**). Results shown as mean ± SD and represent three independent experiments. *p < 0.05, **p < 0.01 when comparing to the condition treated with trypsin at 1 µg/mL every day using two-way ANOVA and Tukey post test. **c**, **d** MOI (0.01 or 0.005) and volume at the time of inoculation (5 or 10 mL) were evaluated, measuring viral titer (**c**) and cell concentration (**d**). Results shown as mean ± SD and represent three independent experiments. *p < 0.05 compared to the condition infected at MOI 0.01–10 mL using two-way ANOVA and Tukey post test.

Firstly, we expected that trypsin addition could have an impact on viral production, as shown in the previous study^35^. So, we compared the effect of four different regimens of trypsin supplementation, varying in trypsin concentration (0.5 or 1 µg/mL in final volume) or in frequency (every day or every 2 days) on Metavac®-RSV virus production over a 4-day period (Fig. 4a, b). Significantly higher titers (3.0 × 10^6^ TCID_50_/mL) were measured at 4 dpi when trypsin was added at 0.5 µg/mL every day or at 1 µg/mL every 2 days compared to 5.3 × 10^5^ TCID_50_/mL when trypsin was added every day at 1 µg/mL (Fig. 4a). In parallel, the cell concentration on microcarriers reached a peak comprised between 9.0 × 10^5^ cells/mL and 10.1 × 10^5^ cells/mL at 1 dpi before decreasing slowly, except when trypsin was added at 0.5 µg/mL daily or at 1 µg/mL every 2 days with a cell density remaining stable during 4 days or declining rapidly below 2.0 × 10^5^ cell/mL at 3 dpi, respectively (Fig. 4b).

Then, we sought to evaluate if a reduced working volume at the time of infection would potentiate viral adsorption depending on the MOI and increase viral production yield or accelerate virus kinetics to achieve a faster peak of virus production. Hence, confluent Vero cell monolayer on microcarriers were infected at an MOI of 0.01 or 0.005 in varying volumes of infection medium supplemented with 1 µg/mL of trypsin, either in 10 mL (as previously) or in 5 mL during 3 h. Viral amplification reached mean maximum virus titers of 3.2 × 10^6^ TCID_50_/mL for MOI 0.01 in 5 mL at 3 dpi or 2.1 × 10^6^ TCID_50_/mL for MOI 0.005 in 5 mL at 4 dpi, whereas a maximum of 2.4 × 10^6^ TCID_50_/mL or 4.4 × 10^5^ TCID_50_/mL was achieved with the same MOI conditions in 10 mL, respectively (Fig. 4c). This result suggests that, in TubeSpin®, lowering the MOI leads to a delayed viral production, but reducing volume during the first 3 h of infection increases viral production. In parallel with these observations, starting from a mean cell density of 7.1 × 10^5^ cells/mL, all the conditions of infection led a cell growth that peaked between 11 × 10^5^ cells/mL and 13 × 10^5^ cells/mL 1 dpi before linear decrease of cell concentration until 4 dpi (Fig. 4d).

In conclusion, we identified the best infection parameters to amplify Metavac®-RSV in a 10 mL working volume of Vero cells cultivated on microcarriers (trypsin added every day at 0.5 µg/mL, and half volume at infection), leading to a 2 log_10_ higher production yield compared to the initial inoculum.

### Scale up Metavac®-RSV production from 10 mL to 2 L stirred-tank bioreactor

**Fig. 5:**
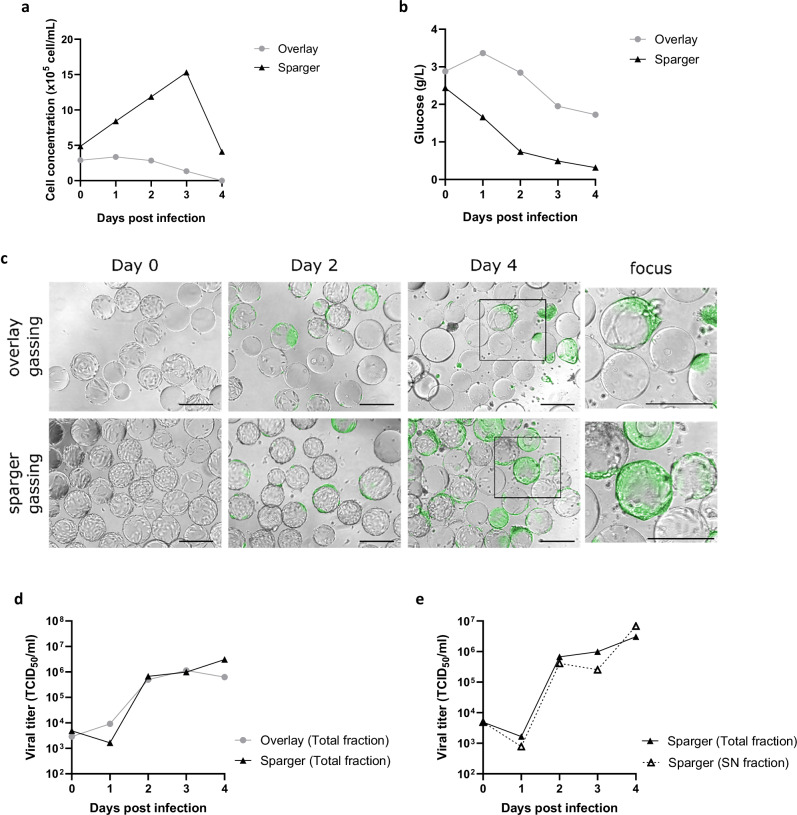
Evaluation of aeration strategies for the production of Metavac®-RSV in 2 L stirred-tank bioreactor. Vero cells were seeded at 1 × 10^5^ cells/mL with Cytodex1 in Univessel SU 2 L. Cells were infected with Metavac®-RSV at MOI 0.01. Throughout 4 days of culture, trypsin was added regularly, and pO_2_ was regulated at 40% by gases added by overlay exchange (gray circle) or sparger pipe (black triangle). **a** Cell concentration, **b** glucose concentration, **c** viral propagation through GFP expression in fluorescent microscopy (scale bar = 250 µm) and **d**, **e** viral titration were monitored every day. Results are shown as the mean of two samples collected in one bioreactor batch.

We then sought to identify the best aeration strategies between overlay or sparger gassing in a 2 L single-use stirred-tank bioreactor with automated control of parameters (temperature, agitation, pH and dissolved oxygen) to scale up viral production of Metavac®-RSV. With a pO_2_ setpoint fixed at 40%, overlay aeration aimed to mimic TubeSpin® cultures with sparger aeration is the most commonly used gassing strategy in large volume bioreactors. Cell growth before and after infection was visibly affected by the gassing strategy. At the time of infection, cells had grown less in the overlay gassing than in the sparger gassing condition, with only 2.9 × 10^5^ cell/mL compared to 5.0 × 10^5^ cell/mL at time 0 (T0), respectively (Fig. 5a). This result is also confirmed by the microscopic observation of cell-free or low cell density microcarriers in the overlay gassing condition at T0 (Fig. 5c). Then, during infection, cells grew only to 3.3 × 10^5^ cell/mL at 1 dpi in the overlay condition, whereas cells continued to grow at high density of 15 × 10^5^ cell/mL at 3 dpi when pO_2_ was regulated with sparger gassing (Fig. 5a). In accordance with these results, glucose consumption was significantly different, as cells grown in overlay condition consumed less glucose than in sparger condition, with 1.7 and 0.3 g/L of remaining glucose at the end of the culture, respectively (Fig. 5b).

Thanks to the follow-up of reporter GFP gene expression in cells infected by Metavac®-RSV, we observed efficient viral propagation in the cells on microcarriers under both conditions. However, visibly less cells were attached on microcarriers at 4 dpi for the overlay gassing condition compared to the sparger gassing condition (Fig. 5c). In the same way, the viral production reached its peak at 3 dpi for the overlay gassing, with 1.14 × 10^6^ TCID_50_/mL, whereas the sparger gassing led to a maximal Metavac®-RSV titer of 3.07 × 10^6^ TCID_50_/mL at 4 dpi (Fig. 5d). In regard to results obtained in static culture, we then compared viral titers obtained in the sparger condition in the supernatant only or in the total fraction (cells and supernatant). Interestingly, it appears that viral titers were similar in the supernatant fraction and in the total fraction, with a titer of 6.83 × 10^6^ TCID_50_/mL at 4 dpi, suggesting that the release of Metavac®-RSV viral particles is more efficient in agitated than in static culture (Fig. 5e). Moreover, we observed and measured viral particles in TEM and we confirmed that their morphology and size were consistent with HMPV (Supplementary Fig. S3a, b).

**Fig. 6:**
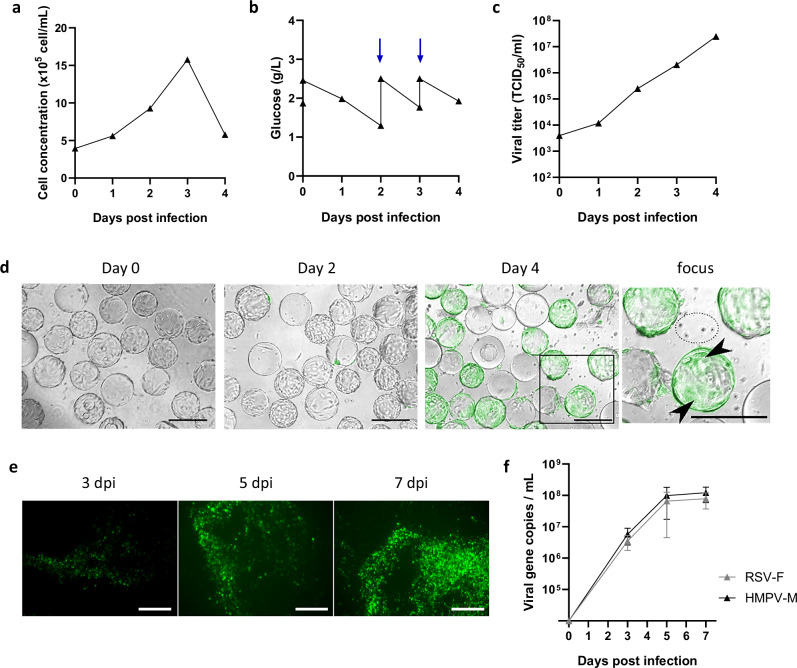
Optimized production of Metavac®-RSV in 2 L stirred-tank bioreactor. **a**, **d** Vero cells were seeded at 1 × 10^5^ cell/mL with Cytodex1 in Univessel SU 2 L. On the infection day, cells were infected with Metavac®-RSV at an MOI of 0.01 after removing of 1 L of the culture medium. Three hours after, the volume was completed with a new medium up to 2 L. Trypsin was added each day at a final concentration of 0.5 µg/mL, and pO_2_ was regulated at 40% by sparger pipe gassing. **a** Cell concentration, **b** glucose concentration (blue arrows represent glucose supplementation), and **c** viral titer were monitored every day. Results are shown as the mean of two samples collected in one bioreactor batch. **d** Viral propagation through GFP expression (scale bar = 250 µm). **e**, **f** Reconstituted human airway epithelium of healthy donors (MucilAir™ nasal, *n* = 3) were infected by apical instillation with Metavac®-RSV harvested from stirred culture at a MOI of 0.1. **e** Images of the GFP+ infected cells were captured using fluorescent microscopy at 3, 5, and 7 dpi (x10 magnification, scale bar = 250 µm). **f** Viral HMPV-M and RSV-F gene copies were quantified by RT-qPCR from apical washes harvested at 3, 5, and 7 dpi. Data were shown as means ± SD.

Finally, after identifying infection parameters on the one hand, and process parameters on the other hand, we combined the best parameters to perform an optimized Metavac®-RSV production batch using a MOI of 0.01 in half total volume during adsorption time, an addition of trypsin every day at 0.5 µg/mL, an aeration strategy through sparger, and a harvest of the supernatant at 4 dpi. As anticipated, these conditions led to a high cell growth until 15.7 × 10^5^ cells/mL at 3 dpi (Fig. 6a) and a pronounced glucose consumption as soon as 2 dpi (Fig. 6b). Before reaching low glucose concentration of 1.8 g/L, we decided to supplement glucose up to 2.5 g/L at 2 and 3 dpi to maintain cell viability and virus production (Fig. 6b). According to the follow-up of reporter (green fluorescent protein GFP) expression, viral propagation was efficient until 4 dpi when we observed cell fusion with large syncytium covering microcarriers and also cell detachment (Fig. 6d). With these optimized conditions, we measured a linear virus amplification through time reaching a maximum titer of 2.48 × 10^7^ TCID_50_/mL at 4 dpi (Fig. 6c). Furthermore, we confirmed that Metavac®-RSV produced in these conditions remains functional and efficiently infects and expresses the RSV-F gene in predictive reconstituted human nasal epithelium model (Fig. 6e, f).

Hereby, we identified and validated the best process parameters that allow amplification of Metavac®-RSV in Vero cells in a 2 L stirred-tank bioreactor. Importantly, we demonstrated the feasibility of the scaled-up production of Metavac®-RSV LAV in Vero cells under industrial manufacturing conditions.

## Discussion

Since approval by the FDA and the EMA, the mitigation of the burden of RSV infection is expected with the two subunit (Arexvy®, GSK and Abrysvo®, Pfizer) and one mRNA (mRESVIA®, Moderna) RSV pre-F protein vaccines in the elderly and newborns (only for Abrysvo®)^12,25–27^. In contrast, no vaccine for RSV is approved for children, while infection causes up to 100,000 deaths in children under the age of 5 around the world, and RSV is the leading cause of infant hospitalization in the United States^5^. Besides supportive measures (administration of oxygen or mechanical ventilation and bronchodilators/corticosteroids), usual countermeasures are monoclonal antibodies targeting the RSV-F protein that can be administered to high-risk (Synagis®, AstraZeneca) or all (Beyfortus®, Sanofi) infants to prevent severe forms of infection^27,53,54^. At the same time, approved anti-HMPV vaccines and countermeasures are still missing despite the high prevalence of this viral infection in children under 5 years of age^7,11,55,56^.

Ongoing development of pediatric vaccines against pneumoviruses has just come to a halt as two clinical trials with experimental RSV or combined RSV-HMPV mRNA vaccines for babies may not only have failed to protect them, but some of them unexpectedly showed higher levels of severe respiratory disease when infants got RSV or another respiratory virus^57^. As happened in the 1960s during the clinical evaluation of inactivated vaccine candidate^58,59^, this safety signal prompted the FDA to suspend enrollment of all other trials involving infants (aged under 2 years) or RSV naive children aged 2–5 years and evaluating vaccine candidates presenting or expressing F or other RSV proteins^60,61^. Intranasal delivery of LAV is considered as a strategy of choice for the pediatric population^30^, as it mimics natural viral infection while eliciting not only robust mucosal response to the respiratory virus entry point but also cellular responses without requiring adjuvant^30^. Moreover, IN-delivered LAVs offer several advantages over IM-administered vaccines, being noninvasive, and more adapted for mass vaccination. Interestingly, Live Attenuated Influenza Vaccine Flumist® (AstraZeneca), which is administered as a nasal spray, is the only example of a human vaccine to have received approval for self-administration by adults up to 49 years of age. Originally approved in the United States in 2003, it can also be administered by a parent or a caregiver to individuals 2–17 years of age^62^.

We previously presented and described an engineered SH gene-deleted recombinant hyperfusogenic virus (Metavac®-RSV), as a promising bivalent live-attenuated vaccine candidate efficiently co-expressing RSV- and HMPV-F proteins in both reconstructed human airway epithelium and mouse preclinical models. We also reported its property to replicate with reduced lung inflammatory scores in mice and to protect them against both RSV and lethal HMPV challenges while inducing strong IgG and broad RSV and HMPV neutralizing antibody titers^36^.

The development of LAV-based strategies is dependent on highly efficient and scalable permissive cell-based production platforms. Whereas RSV can be produced in Vero cell line, a well-established cell line used for production of FDA-approved polio, rabies, rotavirus and smallpox vaccines^39,40^, HMPV tends to be efficiently cultivated on LLC-MK2 cells, which traditionally grow in nonregulatory serum-containing media^1,37,38^. In an effort to reach a manufacturing process scale, we previously characterized the operational parameters to achieve optimal production of functional monovalent Metavac® vaccine candidate in the serum-free avian cell line DuckCelt®-T17 cultivated in suspension (30), but this cell line did not achieve productivity objectives for the bivalent Metavac®-RSV (data not shown).

In this context, we demonstrated that Metavac®-RSV can infect, propagate, and be produced at high titers in a GMP-compliant Vero cell line cultivated on microcarriers in agitated serum-free culture. Importantly, deep RNA sequencing and immunostaining analysis using Palizivumab and HMPV24 mAb showed that Metavac®-RSV produced in Vero cells maintains efficient expression of antigenic F RSV and HMPV proteins after six passages on Vero cells. As expected for RNA viruses, pneumoviruses can adapt to the culture environment through genetic variations^63–66^ and most of the ones we reported were found at a frequency of less than 10% (Table 1 and Supplementary Table S1), confirming acceptable genetic stability of Metavac®-RSV in Vero cells, as previously reported for the LLC-MK2 cell line^36^. Interestingly, very few mutations were observed in the two F protein coding sequences, but none are located in known antigenic epitopes^45–47^ or seem to alter the expression and/or function of RSV and HMPV-F proteins, although it could be further investigated by the use of conformational antibodies.

Furthermore, we assessed properties of Metavac®-RSV produced in Vero cells by using the cotton rat as a reference model for RSV^44,67^ and HMPV^42,43^. This animal model is considered more permissive for RSV infection as it produces 100-fold more viruses than the BALB/c mice, the latter being more resistant to the upper respiratory tract infections^68^. In line with the previous results reported for mice immunized by Metavac®-RSV produced in LLC-MK2 cells^36^, we hence confirm that cotton rats were also protected against both HMPV and RSV challenges after two immunizations with Metavac®-RSV produced in Vero cells. Furthermore, vaccination with Metavac®-RSV induced broad neutralizing antibody and IgG responses in cotton rats, without enhanced pulmonary histopathology or Th2-type cytokine production, in contrast to FI-RSV immunization (Figs. 2, 3)^43,69,70^. Interestingly, we have shown that IN inoculation of Metavac®-RSV induced an increase in pulmonary inflammation and expression of Th1 cytokine IFNγ and chemokines early on (day 5) (Supplementary Figs. 1, 2). This initial elevated expression of cytokines and chemokines after Metavac®-RSV vaccination could contribute to the recruitment of macrophages and T cells into the respiratory tract and the activation of the mucosal immune response. Moreover, the absence of Th2 response after viral challenges suggests that IN vaccination with Metavac®-RSV is not associated with ERD in the experimental conditions tested. Whereas the histopathology score is strongly reduced after two immunizations with Metavac®-RSV and challenges, pulmonary inflammation observed after the first inoculation needs further studies to improve dose, formulation and/or IN delivery of the Metavac®-RSV vaccine candidate.

For clinical development perspectives, this study aimed to identify the optimized operating parameters that enable to efficiently produce Metavac®-RSV LAV candidate within a 2 L single-use stirred-tank bioreactor. Applying the best process parameters, such as multiplicity of infection, trypsin addition, gassing strategy, and glucose supplementation, we obtained favorable conditions for a linear Metavac®-RSV amplification reaching a maximum titer of 2.48 × 10^7^ TCID_50_/mL after 4 days of culture. Importantly, while mechanical cell lysis was required to harvest Metavac®-RSV produced in a static condition of Vero culture, this high production level was reached from the supernatant fraction in the 2 L bioreactor. One hypothesis to explain this divergence is that mechanical stirring, applied to the cells within a bioreactor, leads to shear forces which could facilitate cell excretion of Metavac®-RSV^71^. Moreover, viral particles diameter analysis tends to indicate that Metavac®-RSV particles produced in a bioreactor are smaller and more uniform in size than those obtained from static culture (Supplementary Fig. 3). These characteristics of Metavac®-RSV should certainly benefit the downstream process development by facilitating clarification and concentration of the viral particles through successive steps of tangential filtration and chromatography. To further improve the production process, other key metabolites, such as L-glutamine, lactate, or ammonium, could be measured during the process in order to adjust their concentration if necessary. The possibility of a fed-batch or perfusion culture processes could also be assessed to extend the duration of viral harvest and reach higher production yields of the bivalent LAV candidate. We also confirmed that Metavac®-RSV produced from the pilot process efficiently infects and expresses the RSV-F exogenous gene in a predictive reconstituted human nasal epithelium model (Fig. 6e, f), highlighting the functionality of such a bivalent LAV candidate produced in scalable processes.

In conclusion, we confirm the potential of Metavac®-RSV as an advantageous bivalent live-attenuated vaccine candidate which can bring protection against pneumovirus infections. Associated with an efficient, scalable upstream production process for industrial manufacturing, Metavac®-RSV is a new promising option to protect children and at-risk young adults who need more appropriate strategies of vaccination.

## Methods

### Cells and viruses

Metavac®-RSV is a recombinant bivalent live-attenuated vaccine candidate generated via reverse genetics, as previously described^33,36^. Briefly, Metavac®-RSV is a recombinant HMPV A1 C-85473 attenuated by deletion of SH gene and expressing a GFP reporter gene and a RSV-F gene that was inserted into the viral genome between F and M2 genes.

LLC-MK2 cells (ATCC CCL-7) were maintained in minimal essential medium (MEM, Life Technologies) supplemented with 10% fetal bovine serum (Wisent, St. Bruno, QC, Canada) and 1% penicillin/streptomycin (10,000 U/mL). To establish initial viral seed batch, Metavac®-RSV was rescued and amplified by five serial passages on LLC-MK2 cell monolayers in infection medium (OptiMEM (Gibco, Thermo Fisher Scientific, Waltham, MA, USA) supplemented with 1% penicillin/streptomycin and 0.5 µg/mL acetylated trypsin (T6763, Sigma-Aldrich, Saint Louis, MO, USA), following established protocols^36^. Initial viral stocks were titrated as 50% tissue culture infectious doses (TCID_50_), as previously described^33,35,72^. HEP-2 (ATCC CCL-23) cells were cultivated in MEM medium supplemented with 5% FBS, 1% Pen/Strep, and 2% L-Glu.

Vero cells (Vero WHO 88020401, Nuvonis Technologies GmbH, Vienna, Austria) were maintained in serum-free medium OptiPRO SFM (Gibco, USA) supplemented with 4 mM GlutaMAX (Gibco, USA) in a T175 flask at 37 °C and 5% CO_2_^73^. Metavac®-RSV production on Vero cells was performed in infection medium (OptiPRO SFM (Gibco, Thermo Fisher Scientific, Waltham, MA, USA) supplemented with 4 mM GlutaMAX (Gibco, USA) and 0.5 µg/mL acetylated trypsin (T6763, Sigma-Aldrich, Saint Louis, MO, USA).

### In vitro replicative assay

Confluent LLC-MK2 or Vero cells monolayers in 24-well plates were washed with PBS 1X and infected with an initial stock of Metavac®-RSV at a multiplicity of infection (MOI) of 0.01 in infection media. After an adsorption time of 1.5 h at 37 °C, the infection media was removed and replaced by fresh infection medium supplemented with acetylated trypsin. Trypsin was added at 2 and 4 dpi at 0.5 µg/mL. Samples of supernatants or scraped infected cells and supernatants (total fraction) were collected every 24 h for a duration of 7 days in three independent wells to measure infectious titers by endpoint TCID_50_ titrations. Images of representative cytopathic effects were taken daily using bright-field and fluorescent microscopy (10x magnification, EVOS M5000 Cell Imaging System, Invitrogen, Thermo Fisher Scientific, Waltham, MA, USA).

### Serial passages and viral genome sequencing

Six serial passages of Metavac®-RSV (P + 1 to P + 6) were completed by infection of Vero cells monolayers in 24-well plates at low MOI (10^−3^-10^−4^) in fresh infection medium. The initial viral seed batch of Metavac®-RSV was used for infection of the first passage, and supernatants harvested after 7 days of production were then used for the following successive passages. Viral RNA sequencing was made from samples of supernatant of selected passages P + 1, P + 4, and P + 6. Library preparation was performed from the RNA extracted from samples using the Illumina Stranded Total RNA Prep with Ribo-Zero Plus kit, following the manufacturer’s recommendations. The prepared libraries were sequenced on an Illumina NovaSeq platform, using a 2 × 150 bp paired-end configuration.

The reference proteome was constructed through the combination of proteins extracted from the HMPV genome (OL794355.1, minus SH) obtained from NCBI, supplemented with GFP protein (ADQ48006.1) and RSV-F protein (P03420.1). For contamination detection and removal, two distinct Kraken2 databases were employed. The bacterial and archaeal database was built from NCBI on August 2, 2024. Additionally, a database of vertebrate model organisms was constructed on September 18, 2024, incorporating genomic assemblies from multiple model organisms (GCF_015476345.1, GCF_002263795.3, GCA_018104995.1, GCF_011100685.1, GCF_001704415.2, GCF_034190915.1, GCF_015252025.1, GCF_000002035.6, GCF_018350175.1, GCF_016699485.2, GCF_000001405.40, GCF_037993035.1, GCF_017639785.1, GCF_000001635.27, GCF_011764305.1, GCF_009806435.1, GCF_016772045.2, GCF_036323735.1, GCA_004025045.1, GCF_000003025.6). The processing of raw fastq files was performed using the nexomis/viral-assembly nextflow^74^ pipeline (version 1.2.0). Briefly, read trimming was conducted using fastp (PMID: 30423086) with the pipeline default parameters, followed by an iterative classification process using Kraken2^75^ to eliminate potential host and bacterial contamination. The pipeline retained only unclassified reads for further analysis. Trimmed reads were also mapped to the Metavac®-RSV sequence using Bowtie2^76^ to identify anchored reads. These anchored reads were then merged with the unclassified reads to generate a set of cleaned reads. De novo assembly was performed on the cleaned reads using SPAdes^77^ in “rnaviral” mode^78^. The resulting contigs were evaluated through reference proteome mapping using miniprot^79^, with the contig showing maximum similarity, with the maximum number of proteins being selected for annotation and reorientation as necessary.

Variant analysis was conducted using the nexomis/viral-variant pipeline (version 1.1.1) with default parameters. Cleaned reads were mapped to the Metavac®-RSV sequence reference using BWA-MEM^80^, followed by duplicate marking using Picard tools (“Picard Toolkit.” 2019. Broad Institute, GitHub Repository. https://broadinstitute.github.io/picard/; Broad Institute). Alignment files underwent local realignment using ABRA2^81^, and variants were subsequently called using sav_call (https://github.com/nexomis/sav_call). The final variant analysis incorporated sequence-specific annotation and filtering through the “call.py” script from the sav_call project (Commit: 822331 d). To ensure high-confidence variants, stringent filtering criteria were applied, with indels below 10% frequency and SNPs below 3% frequency being excluded from the final analysis.

### Confocal microscopy

LLC-MK2 cells grown on Lab-Tek II chamber slides (Thermo Fisher Scientific) were infected with a MOI of 0.05 of P + 1 or P + 6 Metavac®-RSV produced on Vero cells. After 3 days of infection, infected cells were fixed with 4% paraformaldehyde in PBS 1X, washed and stained with anti-RSV-F Palivizumab (Synagis®, AstraZeneca) and anti-HMPV-F mAb (HMPV24, Abcam ab94800) antibodies used as primary antibodies in PBS-T at 1:5000 and 1:500 dilutions, as previously described^36^. Nuclei were counterstained with DNA-binding fluorochrome 4,6-diamidinon-2-phenylindole (DAPI, Invitrogen). After staining, the coverslips were mounted with Fluoromount G (Cliniscience) and images were taken using a confocal inverted microscope (Leica Confocal SP5 X), then processed by ImageJ software.

### Animal studies

Viral stock of Metavac® and Metavac®-RSV were produced on static Vero cells cultured in 24-well plates. Total fraction (cell and supernatant) was harvested, clarified, and concentrated by ultracentrifugation, as previously described^36^. Viral stocks for animal studies were titrated in PFU/mL. For the vaccination study, 6–8 week-old *Sigmodon hispidus* female and male cotton rats (Sigmovir Biosystems, Inc., Rockville MD), randomly housed in groups of 5 per cage (included three females and two males) were immunized twice with a 28-day interval via intranasal (IN) route with 3.7 × 10^5^ PFU of Metavac®-RSV, or Metavac® and infected 3 weeks after the second immunization with 10^5^ PFU of RSV A/A2 (RSV, ATCC, Manassas, VA) or HMPV TN/94-49/A2 (HMPV^42^). As control groups, cotton rats were inoculated IN with PBS twice (on days 0 and 28) or infected IN with 10^5^ PFU of RSV or HMPV once (on day 0) and challenged with 10^5^ PFU of RSV or HMPV 7 weeks later. Additional control groups were immunized twice with a 28-day interval IM with formalin-inactivated (FI)-RSV (Lot#100) or FI-HMPV, both diluted 1:100 in PBS, and challenged 3 weeks later with RSV or HMPV. All animals were sacrificed 5 days post-challenge (day 54 of the study) to harvest nasal tissues for viral titration and lungs for viral titration, histopathology, and qPCR analysis. Cotton rats (*n* = 5) were eyebled for serum collection before the first and the second immunization and before the challenge (at day 0, 28, and 49). Sera were used to validate initial seronegative status and to measure neutralizing antibodies via neutralization assays and IgG titers via ELISA assays. To assess replication of Metavac®-RSV in the cotton rat model and measure inflammatory response to immunization, an additional group of five animals was inoculated IN with 3.7 × 10^5^ PFU of Metavac®-RSV and sacrificed 5 days later for collection of nasal tissues for viral titration and lungs for viral titration, histopathology, and qPCR analysis. For manipulation of live animals (e.g., IN administration and blood collection), cotton rats were anesthetized via isoflurane inhalation. Euthanasia of animals was performed by carbon dioxide asphyxiation followed by assuring death, according to the guidelines provided in the Guide (2011) and the American Veterinary Medical Association (AVMA) Guidelines for the Euthanasia of Animals: 2020 edition.

### Ethics

Animal study was performed according with the National Institutes of Health guidelines and Sigmovir Institutional Animal Care and Use Committee’s approved animal study protocol (IACUC Protocol #15).

### Neutralization assays

To evaluate the production of a neutralizing antibody response, heat inactivated sera samples were diluted 1:10 and serially diluted further 1:4. Diluted samples were incubated with 25-50 PFU of RSV A (RSV A2, lot #102313 SSM), RSV B (RSV B 18537, ATCC VR-1580, lot #032417 SSM), or HMPV A (HMPV TN/94-49/A2, lot #030116 SSM) for 1 h at room temperature and inoculated in duplicates onto confluent HEp-2 for RSV, or LLC-MK2 for HMPV. After 2 h incubation at 37 °C in a 5% CO_2_ incubator, the wells were overlaid with 0.75% methylcellulose medium and incubated for 5 days for RSV A, and 7 days for RSV B or for HMPV A.

To read RSV neutralizing antibodies titers, the overlay was removed, and the cells were fixed with 0.1% crystal violet stain and then rinsed and air-dried. For HMPV, the overlay was removed, the cells were fixed with acetone/methanol for 1 h, air-dried, and immuno-stained using anti-HMPV antibody (Curia Biologics, DS7, custom-made), followed by HRP-conjugated secondary antibody (Jackson Immuno Research, 109-035-088). An AEC Chromogen detection solution (Sigma-Aldrich, AEC101) was added to each well and incubated at room temperature for 30 min. Visible RSV or HMPV plaques were counted. The corresponding neutralizing antibody titers were determined at the 60% reduction endpoint of the virus control using the software “plqrd.manual.entry” (Sigmovir Biosystems, Inc).

### IgG quantification

Purified RSV A2 F protein (Sino Biological, 11049) or methanol/acetone fixed HMPV-infected LLC-MK2 cells (in-house, lot #041922) were coated onto a 96-well ELISA plate overnight. The next day, plates were incubated in blocking solution for 1 h at room temperature and subsequently washed. Diluted sera (1:500 in duplicates) along with the positive and negative controls (in-house naive or hyperimmune sera) were added to the wells and incubated at room temperature for 1 h. After washing the plates, chicken-anti-cotton rat IgG-HRP (Immunology Consultants Laboratory Inc., CCOT-25P) diluted 1:20,000 was added to all the wells and incubated for 30 min at room temperature. TMB substrate solution was added to all the wells and incubated at room temperature for 15 min before TMB-Stop solution was added to all of the wells. Optical density (OD) at 450 nm was recorded, and the geometric mean of the duplicate wells was calculated. OD means were then converted to arbitrary units and normalized on the non-vaccinated group for representation.

### Viral titrations

To evaluate viral titers in the respiratory airways of challenged animals, lung and nose tissues were homogenized and samples were clarified by centrifugation and diluted in EMEM. Confluent LLC-MK2 monolayers for HMPV titration or Hep-2 monolayers for RSV titration were infected in duplicates with diluted homogenates in 24-well plates and incubated 2 h at 37 °C in a 5% CO_2_ incubator. The wells were overlaid with 0.75% methylcellulose medium. For RSV titration, after 5 days of incubation, the overlay was removed and the infected Hep-2 were fixed for 2 h with 0.1% crystal violet stain, then rinsed and air-dried. For HMPV titration, after 7 days of incubation, the overlays were removed, the infected LLC-MK2 were fixed for 1 h with acetone and methanol and air-dried for immunostaining as described above. Visible plaques were counted, and viral titers were expressed as plaque-forming units per gram of tissue. Viral titers were calculated as geometric mean ± standard error.

### Histopathology analysis

To measure pulmonary inflammation, lungs were dissected and inflated with 10% neutral buffered formalin to their normal volume, and then immersed in the same fixative solution. Following fixation, the lungs were embedded in paraffin, sectioned, and stained with hematoxylin and eosin (H&E). Slides were scored blind on a 0-4 severity scale for each of the four parameters of pulmonary inflammation evaluated: peribronchiolitis, perivasculitis, interstitial pneumonia, and alveolitis. The scores were subsequently converted to a 0–100% histopathology scale and cumulated for graphical representation.

### Cytokines quantification

Cytokines quantification was performed by RT-qPCR from total RNA extracted from lung homogenate (flash frozen lung lingular lobes). Total RNA was extracted using the RNeasy purification kit (Qiagen). One μg of total RNA was used to prepare cDNA using Super Script II RT (Invitrogen) and oligo dT primer (1 μl, Invitrogen). For real-time PCR reactions, the Bio-Rad iQTM SYBR Green Supermix were used in a final volume of 25 μl, with final primer concentrations of 0.5 μM. Reactions were set up in duplicates in 96-well trays. Amplifications were performed on a Bio-Rad iCycler for one cycle of 95 °C for 3 min, followed by 40 cycles of 95 °C for 10 s, 60 °C for 10 s, and 72 °C for 15 s. The baseline cycles and cycle threshold (Ct) were calculated by the iQ5 software in the PCR baseline-subtracted curve fit mode. Relative quantitation of DNA was applied to all samples. The standard curves were developed using serially diluted cDNA samples, most enriched in the transcript of interest. The Ct values were plotted against log_10_ cDNA dilution factor. These curves were used to convert the Ct values obtained for different samples to relative expression units. These relative expression units were then normalized to the level of β-actin mRNA (“housekeeping gene”) expressed in the corresponding sample.

### Agitated cultures for viral production

Agitated cultures of Vero cells were realized in TubeSpin® 50 mL (TPP®) incubated in a Kühner incubator (ISF1-X, Kühner, Birsfelden, Switzerland) at 37 °C and 5% CO_2_ or in 2 L single-use stirred-tank bioreactor (Univessel® SU, Sartorius, Göttingen, Germany), with a temperature of 37 °C, a pH of 7.2, and a dissolved oxygen (pO_2_) of 40% monitored by Biostat B controller (Sartorius, Göttingen, Germany). Cytodex1 microcarriers (Cytiva, USA) were prepared according to the manufacturer’s instructions, and Vero cells were inoculated on 2 g/L Cytodex1 microcarriers at 1 × 10^5^ cells/mL in a half final volume of culture medium OptiPro SFM supplemented with GlutaMAX (half volume meaning 5 mL in TubeSpin® 50 mL or 1 L in Univessel® SU bioreactor). To protect cells from shear stress, 0.1% Pluronic® F68 (Sigma-Aldrich Co., MO, USA) was added to the culture medium. The following day, fresh media was added up to a final volume of 10 mL in TubeSpin® or 2 L in bioreactor and cells were cultivated for 4 days before virus inoculation.

For trypsin optimization in TubeSpin®, 5 mL of culture medium was removed and replaced by 5 mL of Metavac®-RSV at MOI 0.01 in fresh medium with acetylated trypsin at 0.5 or 1 µg/mL. Depending on the conditions, trypsin was then added to the medium every day or every 2 days at the same final concentration. For MOI and volume at infection optimization in TubeSpin®, 5 mL of culture medium was removed the day of infection and replaced by 5 mL of Metavac®-RSV at MOI 0.005 or 0.01 in fresh medium with acetylated trypsin at 1 µg/mL. Alternatively, 5 mL of culture medium was removed the day of infection, Metavac®-RSV inoculation at MOI 0.005 or 0.01 was made in this volume and incubated for 3 h before addition of 5 mL of fresh infection medium. Trypsin at 1 µg/mL was added to the culture every 2 days during 4 days of viral production. Daily samples of cell and microcarrier suspension were collected to perform bioprocess samples analysis, such as cell count and viral titration.

For aeration strategy evaluation in a bioreactor, Vero cells were cultivated in Univessel® SU set to a temperature of 37 °C and pH of 7.2. pO_2_ was monitored at 40% of air saturation, and O_2_, CO_2_, N_2_, and air gases were added either by overlay or sparger pipe. The setpoint of stirring agitation using two marine-type impellers was set to 70 rpm when overlay gassing, or 85 rpm when gases were added by sparger pipe. Four days after cell seeding, exchange of half of the media was performed, and Metavac®-RSV was inoculated at an MOI of 0.01 in the presence of acetylated trypsin at a final concentration of 1 µg/mL. Trypsin at the same concentration was then added every 2 days during the 4 days of viral production.

Following the best production parameters identified at small-scale and in 2 L Univessel® SU bioreactor, optimized Metavac®-RSV production was conducted in 2 L stirred-tank bioreactor with setpoints of: 37 °C, pH of 7.2, pO_2_ of 40% controlled by sparger gassing and stirring agitation of 85 rpm using two marine-type impellers. Four days after cell seeding, half of the media was removed and Metavac®-RSV was inoculated at an MOI of 0.01 in the presence of acetylated trypsin at a final concentration of 0.5 µg/mL. After 3 h, 1 L of fresh infection media was added to reach a final volume of 2 L. During the 4 days of viral production, trypsin was added every two days. When the glucose concentration dropped below 2 g/L, a glucose supplementation (Glucose solution, Gibco, Thermo Fisher Scientific, Waltham, MA, USA) was performed with a target glucose concentration of 2.5 g/L.

Daily samples of cell and microcarrier suspension were collected to perform bioprocess samples analysis, such as cell count, microscopic observation, and glucose measurement. Samples were also aliquoted and stored at −80 °C for further viral titration.

### Bioprocess samples analysis

To perform cell counting in stirred cultures, 1 mL of cell and microcarrier suspension was collected. After sedimentation, 850 µL of supernatant were discarded. The microcarriers were washed with 1 mL of PBS 1X and 150 µL of trypsin TrypLE 10X (A1217701, Gibco, USA) was mixed to the pellet for 15 min at 37 °C to dissociate cells and microcarriers. Cell counting was then performed with trypan blue on kova® slides (Kova International, California, USA).

Glucose concentration was analysed using One Touch Verio Reflect (LifeScan, USA) from the supernatant.

Bright-field and fluorescent microscopy was performed using EVOS™ M5000 Cell Imaging System (Invitrogen, Thermo Fisher Scientific, Waltham, MA, USA) on the total (cells and supernatant) fraction.

Infectious titers in supernatant or in total fraction were determined using the TCID_50_ method using Vero cells in 96-well plates. Samples were thawed on ice and diluted (supernatant fraction) or thawed on ice and centrifuged to discard the cell pellet before dilution (total fraction). Before infection, the medium was discarded from the cells and samples were diluted in series in the infection medium. Infected plates were incubated for 8 days at 37 °C with 5% CO_2_, and 1% final trypsin was added every two days. TCID_50_ titers were then calculated using the Reed and Muench method.

### Transmission electron microscopy

After production in a 2 L Univessel® SU bioreactor or in static 24-well plates, Metavac®-RSV particles were concentrated by ultracentrifugation, resuspended in NaCl (0.9%) and filtered at 0.45 μm. Viral suspensions were adsorbed on 200-mesh nickel grids coated with formvar-C for 10 min at room temperature. Then, grids with suspensions were colored with Uranyless (Delta Microscopies, Mauressac, France) for 1 min and observed on a transmission electron microscope (Jeol 1400 JEM, Tokyo, Japan) equipped with a Gatan camera (Orius 1000) and Digital Micrograph software. Diameter of Metavac®-RSV particles was measured on transmission electron microscopy pictures (*n* = 58 for bioreactor production and *n* = 30 for static production).

### Infection of reconstituted human airway epithelium

In vitro reconstituted human airway epithelium (HAE), derived from healthy donors’ primary nasal cells (MucilAir™), was purchased from Epithelix (Plan-les-Ouates, Switzerland). HAEs were infected by instillation at the apical side of Metavac®-RSV produced in a bioreactor at an MOI of 0.1. After 2 h of incubation at 37 °C and 5% CO_2_, the inoculum was removed carefully. Infected HAEs were monitored by fluorescent microscopic observation at 3, 5, and 7 dpi using EVOS™ M5000 Cell Imaging System (Invitrogen, Thermo Fisher Scientific, Waltham, MA, USA). At 3, 5, and 7 dpi, apical washes with warm OptiMEM were performed in order to extract viral RNA (QIAamp Viral RNA kit, Qiagen, Hilden, Germany) and viral gene quantification was performed by RT-qPCR targeting the HMPV-M gene or RSV-F gene.

### Statistical analysis

Statistical analyses were performed with GraphPad Prism 10 using *t*-test, one-way or two-way ANOVA with Dunnett posttest or Tukey posttest when comparing to a control group.

## Supplementary information

Supplementary Figures

Supplementary Table

## Data Availability

RNA sequencing analysis supporting the findings of this study are available in Supplementary Table S1. All data sets of RNA sequencing supporting the findings of this study are available in European Nucleotide Archive (ENA - https://www.ebi.ac.uk/ena) under Study Accession number ERA31166782.
