# Supplementary material for: Metavac-RSV mucosal bivalent vaccine candidate protects cotton rats against pneumoviruses and is produced using serum-free cell culture in bioreactor
Source: NPJ Vaccines. 2025 Aug 22;10:202. doi: 10.1038/s41541-025-01231-9 (PMC12373873; doi:10.1038/s41541-025-01231-9)
Supplement: Supplementary file 1 — Supplementary Figures [file 41541_2025_1231_MOESM1_ESM.pdf]

NPJVACCINES - Metavac-RSV mucosal bivalent vaccine candidate protects cotton rats against pneumoviruses and is produced in bioreactor

List of supplementary figures

Figure S1. Virus replication and pulmonary inflammation after IN infection

Figure S2. Pulmonary cytokines levels after viral challenge

Figure S3. Metavac®-RSV produced in 2L stirred-tank bioreactor conserve its morphology

Supplementary figure S1

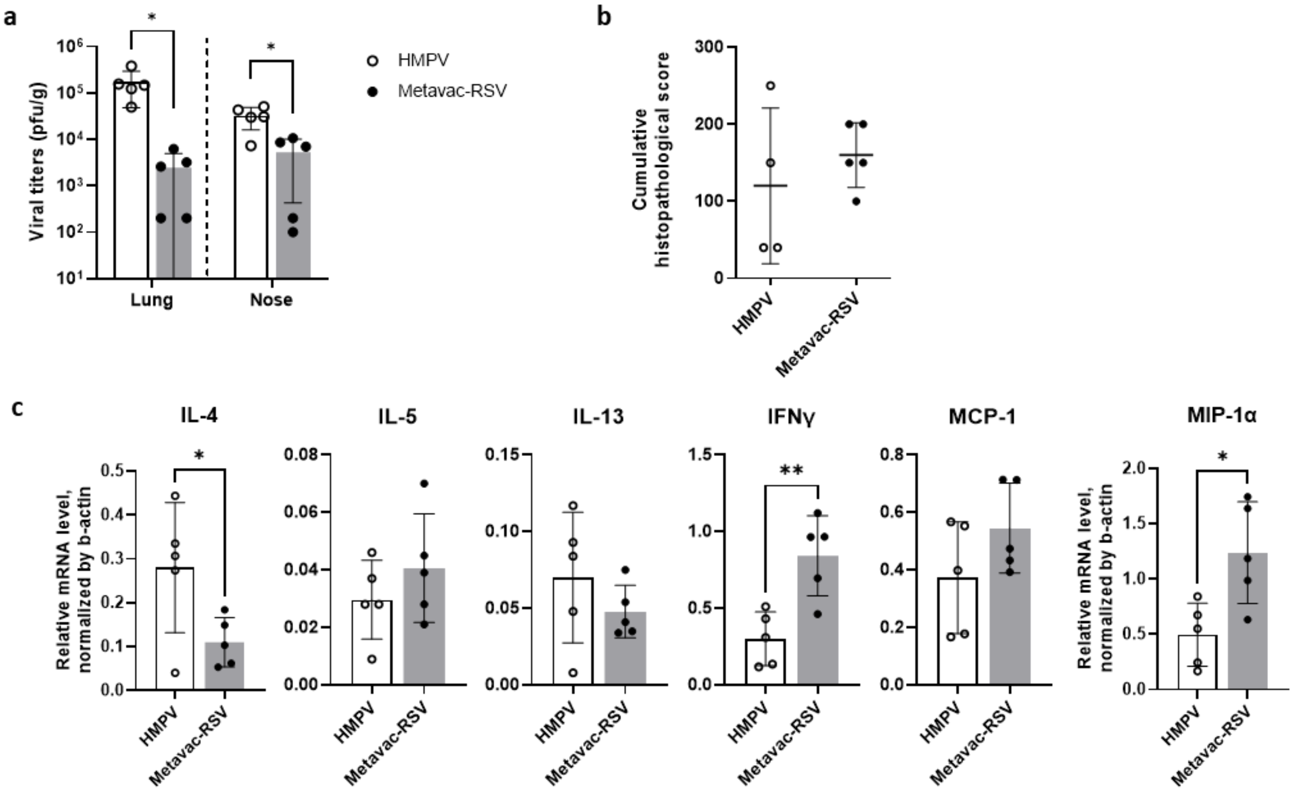

Figure S1. Virus replication and pulmonary inflammation after IN infection.

Nasal tissue and lungs of cotton rats were collected 5 days post infection with 3.7 x 10^5 PFU of Metavac®-RSV or 10^5 PFU of HMPV WT. (**a**) Pulmonary and nasal viral titers were measured in tissues homogenates. . Viral titers were expressed as plaque forming units per gram of tissue and calculated as geometric mean ± SD. *p<0.05 using t-test.
(**b**) Histopathology scores were evaluated in fixed lung tissue and mean cumulative scores are represented. Results are shown as mean ± SD (n=4-5).
(**c**) Cytokines quantification was performed by RT-qPCR from total RNA was extracted from lung homogenate. Relative expression units were normalized to the level of β-actin mRNA (“housekeeping gene”) expressed in the corresponding sample. Results are shown as mean ± SD. *p<0.05, **p<0.01 using t-test.

Supplementary figure S2

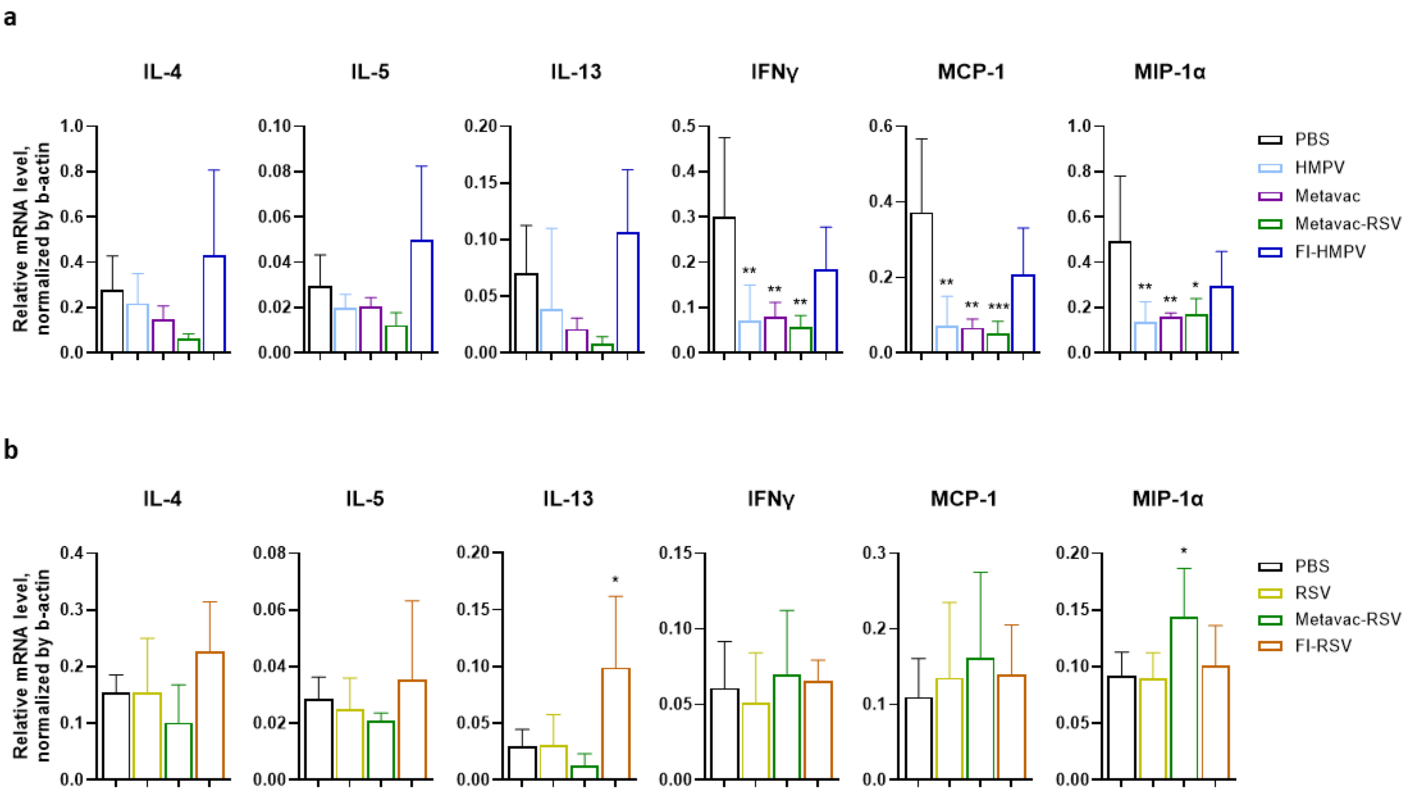

Figure S2. Pulmonary cytokines levels after viral challenge.

Lungs of vaccinated cotton rats were collected 5 days post challenge with HMPV (**a**) or RSV (**b**) for cytokines quantification by RT-qPCR. Results are shown as mean ± SD. *p<0.05, **p<0.01, ***p<0.001 using 1-way ANOVA and Dunnett post-test.

Supplementary figure S3

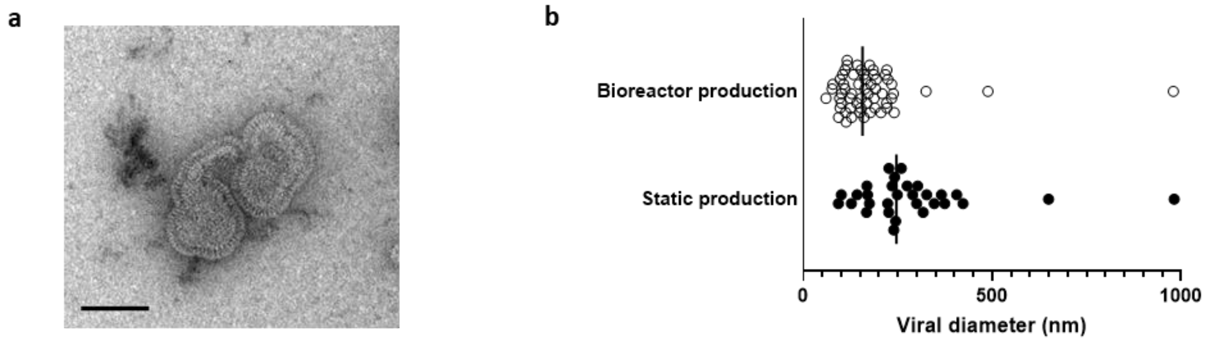

Figure S3. Metavac®-RSV produced in 2L stirred-tank bioreactor conserve its morphology.

a Transmission electron microscopy image of Metavac®-RSV particles produced in bioreactor, concentrated by ultracentrifugation, resuspended in NaCl (0.9%) and filtered at 0.45 µm. Scale bar=100 nm. **b** Diameter of Metavac®-RSV particles produced in bioreactor or in static 24-well plates was measured on transmission electron microscopy pictures (n=58 for bioreactor production and n=30 for static production).
